# O.3.3-4 Development and validation of the obesity-specific decisional balance for Physical Activity questionnaire

**DOI:** 10.1093/eurpub/ckad133.159

**Published:** 2023-09-11

**Authors:** Meggy Hayotte, Thomas Beaudet, Véronique Nègre, Fabienne d'Arripe-Longueville

**Affiliations:** Université Côte d'Azur, LAMHESS, France; Centre Spécialisé Obésité PACA Est, Pôle DARE, Centre Hospitalier Universitaire de Nice, France; Centre Spécialisé Obésité PACA Est, Pôle DARE, Centre Hospitalier Universitaire de Nice, France; Université Côte d'Azur, LAMHESS, France

## Abstract

**Purpose:**

Despite the crucial role of physical activity in obesity management, many patients remain insufficiently physically active. The decisional balance, from the transtheoretical model, represents the facilitators and the barriers associated with behavior change (e.g., engaging in regular physical activity). Studies have identified barriers to and facilitators of physical activity specific to the context of obesity. However, to date, there is no scale to measure them. The objective of this study was to develop and validate a preliminary version of the Obesity-specific Decisional Balance for Physical Activity (O-DBPA).

**Methods:**

A series of studies was conducted with a total sample of 350 adults with obesity (M_age_=44.3, SD = 14.7 years; M_IMC_=44.4, SD = 14.7 kg.m^2^). First, the clarity of the 50 items developed by the expert committee was examined. Second, the dimensionality of the scale was examined through confirmatory factor analyses. Third, reliability tests were performed (i.e., Cronbach's alphas, test-retest reliability). Fourth, theoretical validity of the O-DBPA was examined through its correlations with other theoretically related constructs (i.e., motivation, self-esteem).

**Results:**

Clarity assessment revealed acceptable scores for all items (M = 6.2/7, SD = 1.1). Dimensionality testing led to reduce the scale to 21 items divided into two factors: facilitators of and barriers to physical activity. Each of these factors was composed of three subscales: physical, psychological, and environmental. The confirmatory two-factor model had the best fit indices: χ^2^(166)=323.67, CFI=0.95, TLI=0.93, RMSEA=0.052. Cronbach's alphas were acceptable or marginally acceptable (i.e., [0.61-0.88]). Temporal reliability, measured with a 4-week interval, was satisfactory. The O-DBPA subscales were significantly related to the motivation and self-esteem constructs in the expected directions, attesting to the theoretical validity of the scale.

**Conclusion:**

The O-DBPA, composed of 21 items, presented good psychometric qualities. This new instrument allowed to examine the obesity-specific barriers to and facilitators of physical activity in French-speaking samples. This new scale offers promising clinical and research perspectives for the promotion of physical activity in adults with obesity.

